# Rediscovery and taxonomic placement of *Solanum polyphyllum* Phil. (Solanaceae), a narrow endemic from the Chilean Atacama Desert

**DOI:** 10.3897/phytokeys.156.53703

**Published:** 2020-08-21

**Authors:** Andrés Moreira-Muñoz, Mélica Muñoz-Schick

**Affiliations:** 1 Instituto de Geografía, Pontificia Universidad Católica de Valparaíso, Avenida Brasil 2241, Valparaíso, Chile Pontificia Universidad Católica de Valparaíso Valparaiso Chile; 2 Museo Nacional de Historia Natural, Casilla 787, Santiago, Chile Museo Nacional de Historia Natural Santiago Chile

**Keywords:** Chile, rediscovery, *Regmandra, Solanum*, *Solanum polyphyllum*, *Solanum* sect., Tarapacá Region

## Abstract

Although the original description of *Solanum polyphyllum* Phil. was made in 1891, this species was not seen until it was re-discovered 128 years later in 2019 in the Atacama Desert. Fruits and seeds were previously unknown and a complete description is provided here. This species was not treated in the most recent monograph of Solanum sect. Regmandra, but it should be incorporated in this section due to its glabrous, sessile and entire leaves, which are decurrent onto the stem. Morphologically, *S. polyphyllum* is similar to S. *paposanum*, also of section Regmandra, but differs in the entire leaves (against margins with 4–5 acute lobes in *S. paposanum*) and glabrous leaves (moderately pubescent adaxially and velutinous abaxially in *S. paposanum*). The rediscovery of *S. polyphyllum* at a new locality at the same altitudinal belt as the type, re-affirms its restricted distribution and endemism and supports a potential conservation status as an endangered species.

## Introduction

*Solanum* is a sub-cosmopolitan genus of about 1400 species (Bohs 2005, Weese and Bohs 2007), with the highest diversity in the Andes of South America (Knapp 2002). In Chile, it includes 63 species (44 native and 19 endemic) (Moreira-Muñoz 2011), distributed along an immense latitudinal gradient from arid environments at 17°35'S latitude to hyper-humid southern Chile around 50°S. *Solanum* species are also found in Juan Fernández archipelago, Desventuradas Islands and Easter Island (Rapa Nui). During a recent botanical survey of plant and insect diversity along a transect in the Atacama desert-highlands in 2019, a remarkable specimen of *Solanum* with flowers and fruits was collected.

Rémy (1849) was the first to group and describe Chilean *Solanum* species (18 spp.). Rudolph A. Philippi, the prolific naturalist, described 40 additional species between 1858 and 1895 (e.g. Philippi 1858, 1891, 1895) including *Solanum polyphyllum*. In the second *Flora of Chile*, Reiche (1909) grouped the 50 Chilean *Solanum* species he recognised into six groups. He placed *S. polyphyllum* in his Group II corresponding to section Pachystemonum subsection Dulcamara, in accordance with a manuscript sent to him by the specialist Miss J. Witasek from Vienna (see note in Reiche 1909, pg. 716).

Reiche indicated that his Group II included herbaceous species with markedly winged stems due to the decurrent leaves. In it he included three species: *S. phyllanthum* Cav. (with lobulate leaves, hispid indumentum, now recognised as *S. montanum* L.), *S. herbabona* Reiche (with velutinous, lobulate leaves) and *S. polyphyllum* Phil. (with entire, glabrous leaves). He also included, in his Group III (prismatic stems, sometimes marked by prominent lines) of section Pachystemonum Subsection Dulcamara, the herbaceous species with pinnatifid up to bipinnatifid leaves; here he treated 12 species, six of them currently considered members of section Regmandra (Bennett 2008).

Bennett (2008) included eleven species in his circumscription of section Regmandra, of which nine are native or endemic to Chile, but did not include *S. polyphyllum*. As part of the survey of the flora of Tarapacá, a plant of what appeared to be *Solanum polyphyllum* was found in Compe, on the road to Camiña, in May 2019. That season was relatively wetter than normal, and the vegetation was fully developed and green. These types of greenings or desert blooms associated with the El Niño phenomenon are better known from the southern Atacama, but seem to be a new phenomenon in the northern part of Atacama, in the region of the transition from the driest desert (1900 to 2200 m) to the more humid precordillera and Altiplano. Specific climatic data are sparse, and remote sensing approaches have only recently been undertaken (Chávez et al. 2019). Recollected specimens of the *S. polyphyllum* were compared with the type and the description, and we realised this was a species that had not been collected since 1891. Here we provide a modern description for this rare species, and suggest it belongs in section Regmandra. We also provide a key to the Chilean species of section Regmandra to assist others in distinguishing *S. polyphyllum* in order that it might be found again.

## Taxonomic treatment

### Solanum polyphyllum

Taxon classificationPlantaeSolanalesSolanaceae

Phil., Anales Museo Nacional, Botánica 2: 64, 1891

082921BE-41A1-53B1-A107-FAD03678B429

Figures 1 , 2

#### Type.

Chile. Tarapacá Region: prov. Tarapacá, Pachica, 12 Mar 1885, *C. Rahmer s.n.* (lectotype, designated here: SGO [SGO000004586 acc.#55603]; isolectotypes: B [destroyed, F neg. 2739], CORD [CORD00004258], SGO [SGO000004587 acc.#42779]). Images available via https://plants.jstor.org/.

**Figure 1.**
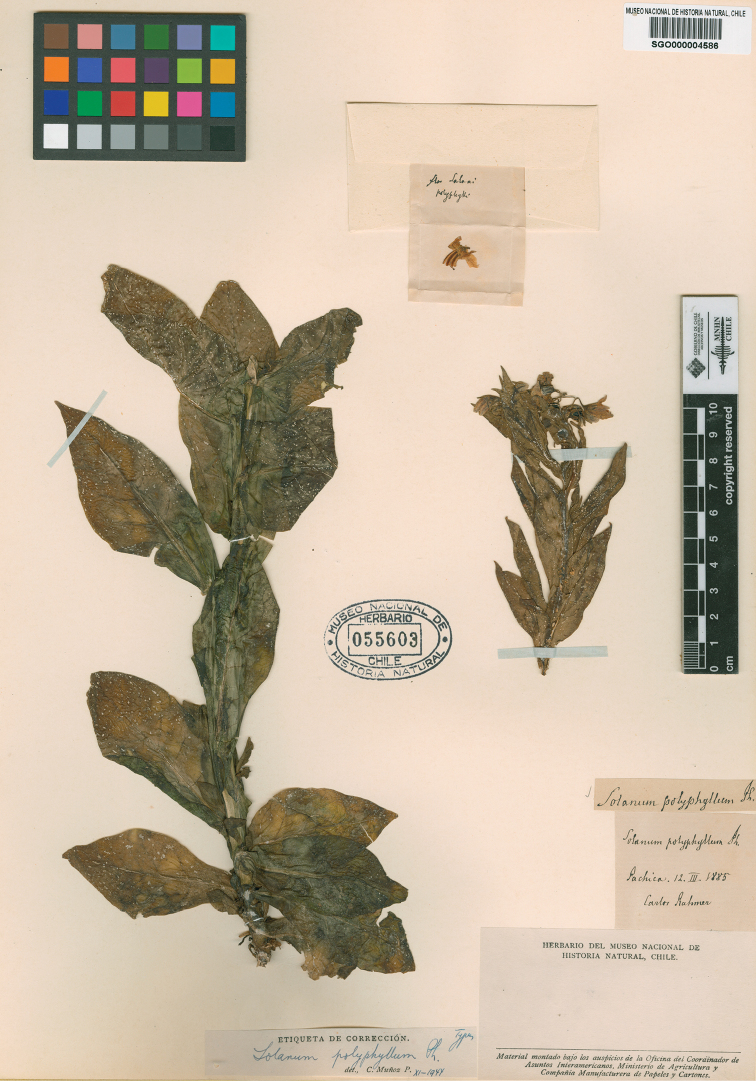
*Solanum polyphyllum* Phil. (lectotype: *C. Rahmer s.n.*, SGO000004586 acc.#55603).

#### Description.

**Figure 2.**
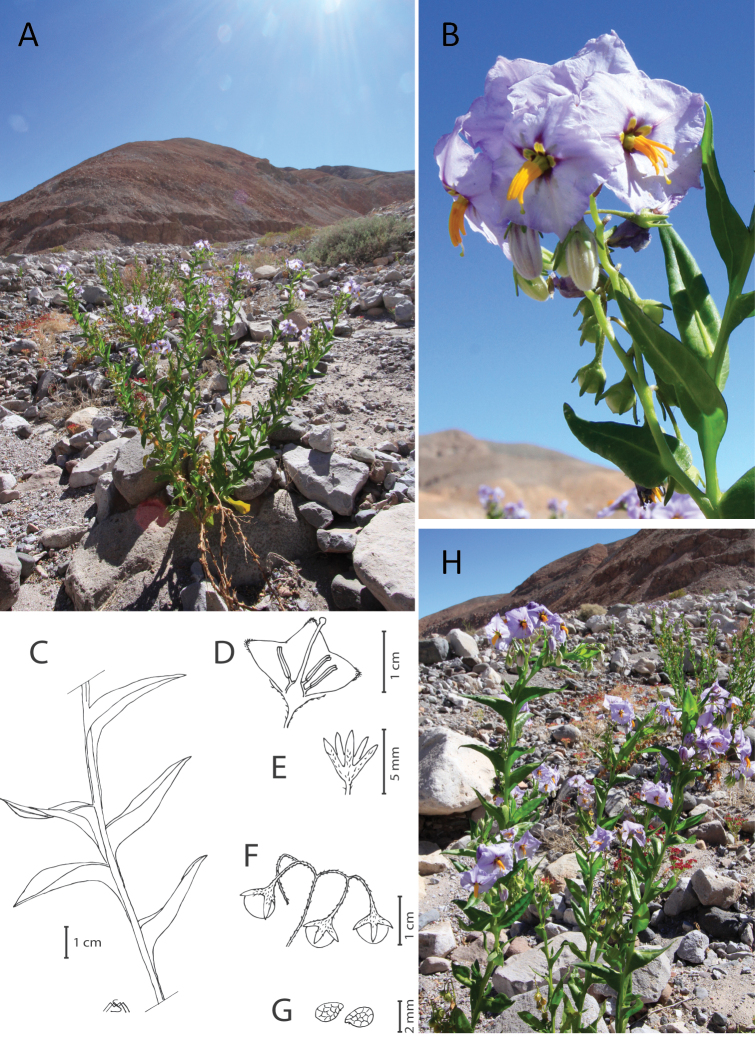
*Solanum polyphyllum* at an alluvial cone at the Tarapacá precordillera **A** habitat **B** flower details **C** leaves **D** flower **E** calyx **F** fruits **G** seeds **H** habit. Photos by A. Moreira-Muñoz, drawings by M. Muñoz-Schick.

Perennial, robust herbaceous plant up to 100 cm tall. *Stems* thick, glabrescent, internodes with a wing up to 3 mm wide. *Leaves* simple, the blades 3–4 (-8) cm long x 2–4 cm wide, ovate-lanceolate, sessile, base decurrent on the winged petiole, shiny or with few thick hairs, with yellow crystals included in the midrib and stems; upper leaves shorter and thinner. In the buds, there is an oval leaf, with a petiole that has some whitish hairs. *Inflorescence* 7–11 cm long, leaf-opposed, with 12–30 flowers; pedicels filiform 10–12 mm long, with few white hairs 0.5 mm long, which continue in the calyx; calyx 4–5 mm long, with a short tube, ¼-1/3 of its length, divided into five almost linear divisions, 1 mm wide. *Corolla* blue, ca. 15 mm in diameter, pentagonal, with five shallow divisions, purple colored at its base forming a star, that is alternated with notorious oblong and yellow-green nectaries, pubescent abaxially mainly towards the apices of the divisions, glabrous adaxially. *Stamens* unequal; anthers differing in size, 4 of 6–9 mm long, 1 shorter of 4–7 mm long, or 3 shorter and 2 longer, tapered, the narrow apex with an elongating pore, the longer anthers curving towards the shorter ones. *Style* curved and longer than the anthers, stigma capitate. *Fruit* 5–6 mm in diameter, a glabrous, globose, shiny green-orange berry. *Seeds* white, ca. 2 mm long, ca.1.5 mm wide, reniform, with a reticulate surface (Fig. 2).

#### Distribution.

Endemic to the Atacama desert of northern Chile; only known from two localities in the precordillera of Tamarugal province, Tarapacá region.

#### Ecology and habitat.

*Solanum polyphyllum* grows between loose rocks of an alluvial cone on the north side of the Camiña river. It corresponds to a plant of 100 cm high, erect stems, very blue flowers and green-orange berries. The population is composed of only a dozen exemplars. The vegetation of the site is an open bush of low coverage (15%). Other species present on the site are: *Cistanthe amarantoides* (Phil.) Carolin ex Hershkovitz (Montiaceae), *Encelia oblongifolia* DC., *Helogyne apaloidea* Nutt. (both Asteraceae), *Malesherbia tenuifolia* D.Don (Passifloraceae), *Huidobria fruticosa* Phil. (Loasaceae), *Allionia incarnata* L. (Nyctaginaceae) and *Exodeconus integrifolius* (Phil.) Axelius (Solanaceae).

#### Conservation status.

The limited representation in herbaria, as well as the low abundance in the field, makes the *Solanum polyphyllum* a candidate for threatened status. Due to its distribution in the two known locations of Compe and Pachica it is Data Deficient, pending further field work. *Solanum polyphyllum* potentially could be found in Isluga National Park but new surveys are required to corroborate its presence in this protected area.

#### Discussion.

In its broadly decurrent leaves on the stem, *S. polyphyllum* is similar to *S. paposanum* Phil., which grows both in Perú and in Chile, from 200–3500 m elevation. It differs from *S. paposanum* in its entire (versus margins with 4–5 acute lobes in *S. paposanum*) and glabrous leaves (versus moderately pubescent adaxially and velutinous abaxially in *S. paposanum*).

*Solanum polyphyllum* was described by Rodulfo Amando Philippi based on specimens collected in Pachica (19°51'58"S, 69°25'56" W, 1622 m alt.) by Carlos Rahmer during a journey to Tarapacá made in 1885. Of this gathering, two duplicates are conserved in SGO. We designate as lectotype SGO000004586 (acc.#55603, Fig. 1), which is very complete and has the handwritten label of R.A. Philippi. The duplicate specimen (SGO000004587, acc.#42779) has a label written by Federico Philippi (son of R.A. Philippi) who also participated in the 1885 expedition.

The rediscovery of this remarkable plant provides an opportunity to promote more intensive fieldwork in the Tarapacá cordillera, at the transition belt between the desert and precordillera vegetation types, where vegetation greening seems to occur more often due to regional climate change. Despite the sparse vegetation that dominates in the Atacama, new botanical discoveries in different plant families are currently happening, as in Asteraceae [*Senecio*] (Calvo and Moreira-Muñoz 2019, 2020), Basellaceae [*Anredera*] (Moreira-Muñoz and Muñoz-Schick 2018), and Solanaceae [*Schizanthus*] (Morales et al. 2020).

#### Additional specimens examined.

**Chile.** Tarapacá Region, Route A-45 towards Camiña, Compe locality, 19°21'9"S, 69°31'18"W, 1950 m alt., 16 May 2019, *A. Moreira 3038* (SGO).

### Key to Chilean species of Solanum section Regmandra (based on Bennett 2008)

**Table:** 

1	Leaf blades pinnatifid or bipinnatifid	**2**
1a	Leaf blades entire, subentire or lobed	**3**
2	Segments of leaves 1 mm wide; corolla 5–7 mm in diameter; Chile: San Ambrosio Island and the coast of northern Chile (Tarapacá, Antofagasta, Atacama Regions)	***S. brachyantherum* Phil.**
2a	Segments of leaves 3 mm wide; corolla 11–22 mm in diameter, northern Chile (Antofagasta, Atacama and Coquimbo Regions)	***S. remyanum* Phil.**
3	Anthers unequal in length	**4**
4	Blades with lobed margins, lobes with a smaller secondary lobe arising obliquely at its upper part, both leaf surfaces densely tomentose, Chile (Atacama, Coquimbo and Valparaíso Regions) on dunes and sandy lomas	***S. trinominum* J.R.Benn.**
4a	Blades with entire, subentire or shallowly crenate margins	5
5	Leaf base cuneate, leaf surface scabrous, Chile (Valparaíso Region) on sandy slopes	***S. indivisum* Witasek ex J.R. Benn.**
5a	Leaf base decurrent	**6**
6	Leaves with scabrous surface; Chile (southern coast of Coquimbo Region) on sandy slopes	***S. coquimbense* J.R. Benn.**
6a	Leaves with glabrous surface; Chile (Tarapacá Region, 1600–1950 m)	***S. polyphyllum* Phil**.
3a	Anthers equal in length	**7**
7	Leaves densely velutinous	**8**
8	Leaf base decurrent on prominently winged petioles, the wings up to 10 mm wide; coast to high altitudes, 3500 m; Chile (Arica and Parinacota to Coquimbo Regions) and Perú	***S. paposanum* Phil.**
8a	Leaf base truncate, leaves usually petiolate, the petioles without or with a wing up to 1 mm wide; Chile (coastal Atacama Region), ca. 800 m	***S. herbabona* Reiche**
7a	Leaves glabrous to moderately pubescent	**9**
9	Inflorescence with 4–10 flowers; leaf margin subentire to deeply lobed, with 2–3 pairs of lobes, the lobe length less than a third of the total leaf width; Chile (coast of Tarapacá) and Perú; on sandy or rocky lomas	***S. montanum* L.**
9a	Inflorescence with 12–40 flowers; leaf margin with 3–5 pairs of lobes, the lobe length a third to a half of the leaf width; Chile (Arica and Parinacota, Tarapacá, Atacama, Coquimbo, Valparaíso, O’Higgins and Biobío Regions) dunes and coastal slopes	***S. pinnatum* Cav.**

## Supplementary Material

XML Treatment for Solanum polyphyllum
